# A novel antioxidant ergothioneine PET radioligand for in vivo imaging applications

**DOI:** 10.1038/s41598-021-97925-w

**Published:** 2021-09-16

**Authors:** William J. Behof, Clayton A. Whitmore, Justin R. Haynes, Adam J. Rosenberg, Mohammed N. Tantawy, Todd E. Peterson, Fiona E. Harrison, Robert B. Beelman, Wellington Pham

**Affiliations:** 1 Vanderbilt University Institute of Imaging Science, Vanderbilt University Medical Center, Nashville, TN 37232 USA; 2 Department of Radiology and Radiological Sciences, Vanderbilt University Medical Center, Nashville, TN 37232 USA; 3 Department of Medicine, Diabetes, Endocrinology and Metabolism, Vanderbilt University Medical Center, Nashville, TN 37232 USA; 4 Department of Food Science, Center for Plant and Mushroom Foods for Health, Penn State University, University Park, PA 16802 USA; 5 Department of Biomedical Engineering, Vanderbilt University, Nashville, TN 37235 USA; 6 Vanderbilt Brain Institute, Vanderbilt University, Nashville, TN 37232 USA; 7 Vanderbilt Ingram Cancer Center, Nashville, TN 37232 USA; 8 Vanderbilt Institute of Chemical Biology, Vanderbilt University, Nashville, TN 37232 USA; 9 Vanderbilt Institute of Nanoscale Science and Engineering, Vanderbilt University, Nashville, TN 37235 USA; 10 Vanderbilt Memory and Alzheimer’s Center, Vanderbilt University Medical Center, Nashville, TN 37212 USA

**Keywords:** Biotechnology, Neuroscience, Biomarkers, Chemistry

## Abstract

Ergothioneine (ERGO) is a rare amino acid mostly found in fungi, including mushrooms, with recognized antioxidant activity to protect tissues from damage by reactive oxygen species (ROS) components. Prior to this publication, the biodistribution of ERGO has been performed solely in vitro using extracted tissues. The aim of this study was to develop a feasible chemistry for the synthesis of an ERGO PET radioligand, [^11^C]ERGO, to facilitate in vivo study. The radioligand probe was synthesized with identical structure to ERGO by employing an orthogonal protection/deprotection approach. [^11^C]methylation of the precursor was performed via [^11^C]CH_3_OTf to provide [^11^C]ERGO radioligand. The [^11^C]ERGO was isolated by RP-HPLC with a molar activity of 690 TBq/mmol. To demonstrate the biodistribution of the radioligand, we administered approximately 37 MBq/0.1 mL in 5XFAD mice, a mouse model of Alzheimer’s disease via the tail vein. The distribution of ERGO in the brain was monitored using 90-min dynamic PET scans. The delivery and specific retention of [^11^C]ERGO in an LPS-mediated neuroinflammation mouse model was also demonstrated. For the pharmacokinetic study, the concentration of the compound in the serum started to decrease 10 min after injection while starting to distribute in other peripheral tissues. In particular, a significant amount of the compound was found in the eyes and small intestine. The radioligand was also distributed in several regions of the brain of 5XFAD mice, and the signal remained strong 30 min post-injection. This is the first time the biodistribution of this antioxidant and rare amino acid has been demonstrated in a preclinical mouse model in a highly sensitive and non-invasive manner.

## Introduction

**Figure 1:**
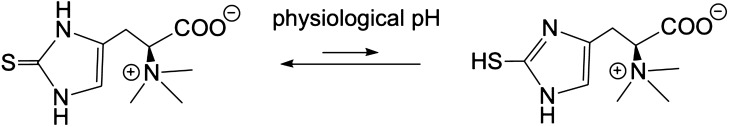
Tautomerized isoforms of ERGO at physiological pH.

L-ergothioneine (ERGO) is a food-derived hydrophilic antioxidant available in fungi and various bacteria, but not in animals or higher plants^1^. It has been known as an antioxidant since its discovery a century ago from fungi of the genus claviceps purpurea^2–4^. Chemically, this rare betaine-based amino acid has a similar chemical structure to histidine but with the presence of a sulfhydryl moiety on the imidazole ring. The molecule exists as a tautomeric form between thioketone and thiol derivatives (Fig. 1), albeit the former predominates at physiological pH^5^. Because plants and animals cannot produce ERGO, it must be obtained from the diet. Mushrooms, in particular are a rich source of ERGO^6–10^. As an antioxidant agent ERGO is capable of preventing cell and tissue damage, a key contributor to aging, by protecting against free radicals and oxidative stress^11–13^. Its acquired adaptive antioxidant capability for the protection of injured tissues^2^ could be the reason for the observations that the highest concentration of ERGO are usually found in the red blood cells of old-age individuals^14^, brain^15^, liver^16^, kidney^16^, ocular tissues^17^, and injured tissues^18^. Additional evidence suggests that ERGO could target mitochondria and dampen the excess of mitochondria-specific ROS in response to oxidative stress^19^.

ERGO is also implicated in a number of neurological pathways^20–23^. Substantial research data indicate that ERGO is a physiological antioxidant cytoprotectant^4^. Protection against cytotoxicity elicited by Cu (II), hydrogen peroxide, iron, and sodium nitrite^16,24–26^ is derived from the conspicuous affinity of ERGO for metal cations, such as Fe and Cu, permitting capture and neutralization of associated radicals^27^. It has been demonstrated that ERGO concentration decreases significantly with age, and markedly lower levels were found in individuals with mild cognitive impairment compared to the controls, supporting the potential for ERGO deficiency to act as a risk factor for neurodegeneration^28^. It is worthwhile to mention that ERGO does not permeate the blood–brain barrier (BBB); its uptake in cells is mediated by an OCTN1 (organic cation transporter novel, type 1) receptor^29–32^. Other studies have shown that ERGO can protect neurons both in vitro and in vivo against a spectrum of stressors^33,34^. Taken altogether, these data strongly suggest that ERGO is involved in healthy aging, serving as a “longevity vitamin”^6,35^. The term “vitamin” is used in here since ERGO is a micronutrient, that the body does not need a lot of to function, but the material needs to be constantly available by uptake from an external source such as foods or supplements and it therefore does fit into that category.

To date, all preclinical studies had relied solely on in vitro analysis of ERGO using analytical and bioassay methods for analyzing the ERGO distribution from extracted tissues^1,36,37^. The same holds true for clinical studies to validate the benefit of ERGO consumption in the diet; serum samples were collected for assessing the ERGO levels^38,39^. This necessitated the development of an ERGO probe to facilitate non-invasive, real-time, and repeated imaging of ERGO distribution and pharmacokinetics in preclinical and clinical settings. To this end, we report herein for the first time on the development of a [^11^C]ERGO radioligand. Further, we demonstrated that this antioxidant could be distributed and retained in the brain of a mouse model of Alzheimer’s disease, assuming due to the inflammation in the local areas. And that observation was further confirmed using a mouse model of LPS-induced neuroinflammation. The proof-of-principle PET imaging in this study showed that ERGO is distributed across several subregions in the brains, albeit with much higher retention in pathological brains than normal counterparts. Interestingly, the whole-body biodistribution study showed that ERGO is also distributed significantly in the eyes and small intestine.

## Results

### Retrosynthesis analysis and development of precursor

**Figure 2:**
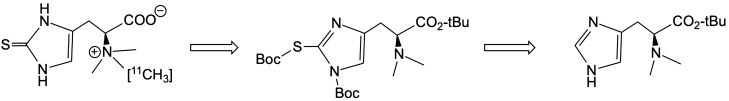
Retrosynthetic analysis of a precursor for generating a [^11^C]ERGO PET radioligand.

Retrosynthetic analysis of [^11^C]CH_3_ labeled ERGO suggests the precursor should have the thiol-histidine molecule with the dimethylamine site available for labeling, while the rest of other functional groups should be uniformly blocked with acid-labile protection groups (Fig. 2). They can thus be removed simultaneously as quickly as possible given the short half-life of [^11^C]CH_3_. The next major transformation involves the insertion of the thiol group into the imidazole ring.

**Figure 3:**
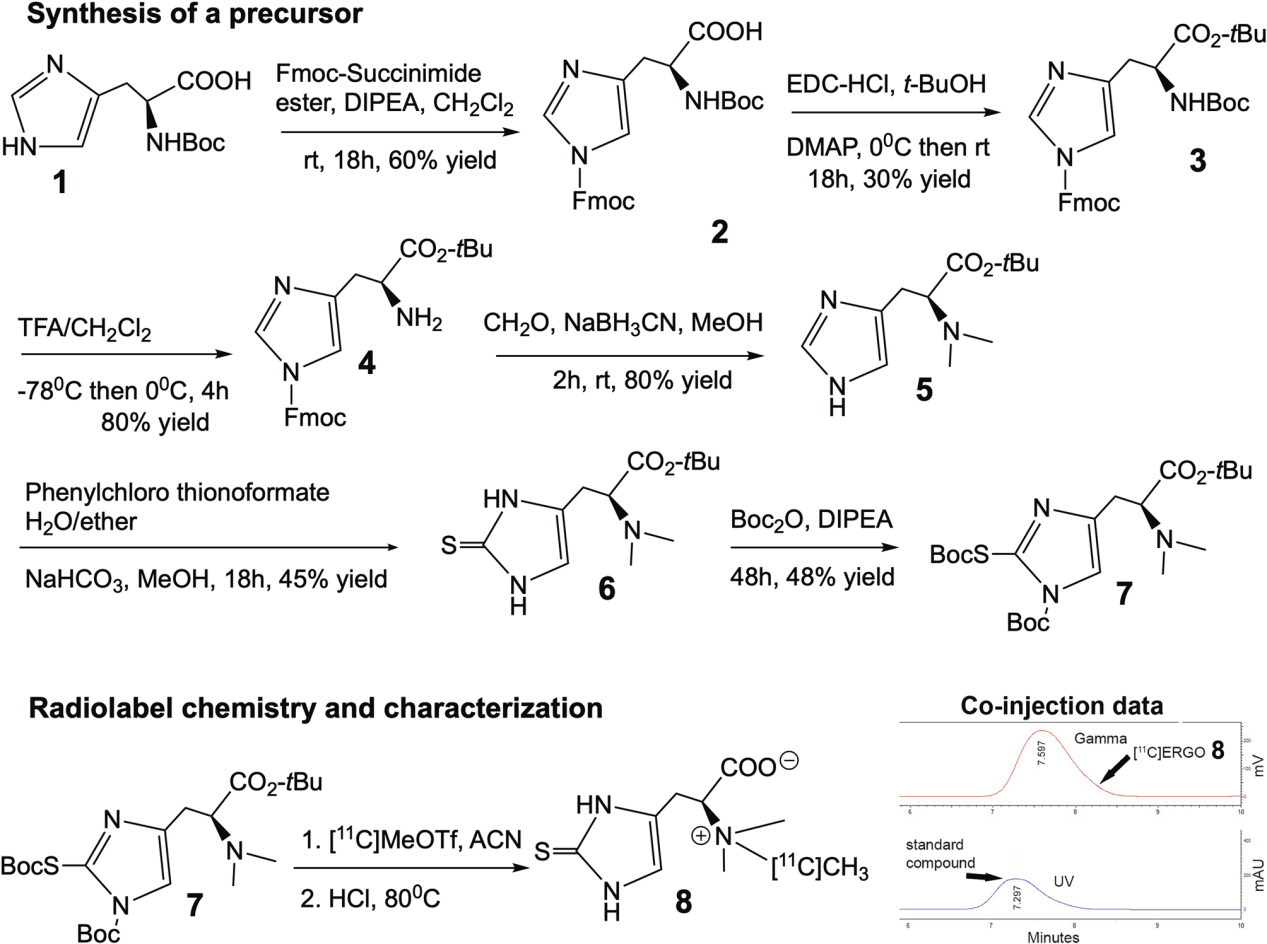
Scheme of [^11^C]ERGO PET radioligand synthesis and characterization.

Figure 3 describes the successful synthesis of [^11^C]ERGO PET radiotracer **8**. The preparation of intermediate **4** would take three steps starting from protected histidine **1** to set the stage for the crucial synthesis of compound **5**. The formation of key intermediate **5** via dimethylation of the α-amino group was achieved selectively after orthogonal protection of the other functional groups. When the solution of **5** in a mixture of water and diethyl ether was treated with O-phenylchloro thionoformate in the presence of sodium bicarbonate and stirred overnight at room temperature to furnish thioketone intermediate **6**. Finally, the precursor **7** was synthesized by treating compound **6** with Boc anhydride to ensure all the active functional group are capped with acid labile groups.

With the precursor available, next, we labeled the ERGO precursor **7** with [^11^C]CH_3_ using a commercial automated radiosynthesis module (GE TRACERlab FXc-Pro). The [^11^C]CO_2_ was converted to [^11^C]MeOTf using the standard reaction conditions, reacted with precursor **7** at 80 °C, and subsequently deprotected under acidic conditions to yield [^11^C]ERGO **8** (Supplementary data, Fig. S1). The [^11^C]ERGO was pH adjusted by passage through an ion-retardation resin and used without further purification. The product was produced with an excellent molar activity of 690 TBq/mmol.

### In vivo dynamic PET scanning of [^11^C]ERGO biodistribution in the brain

**Figure 4:**
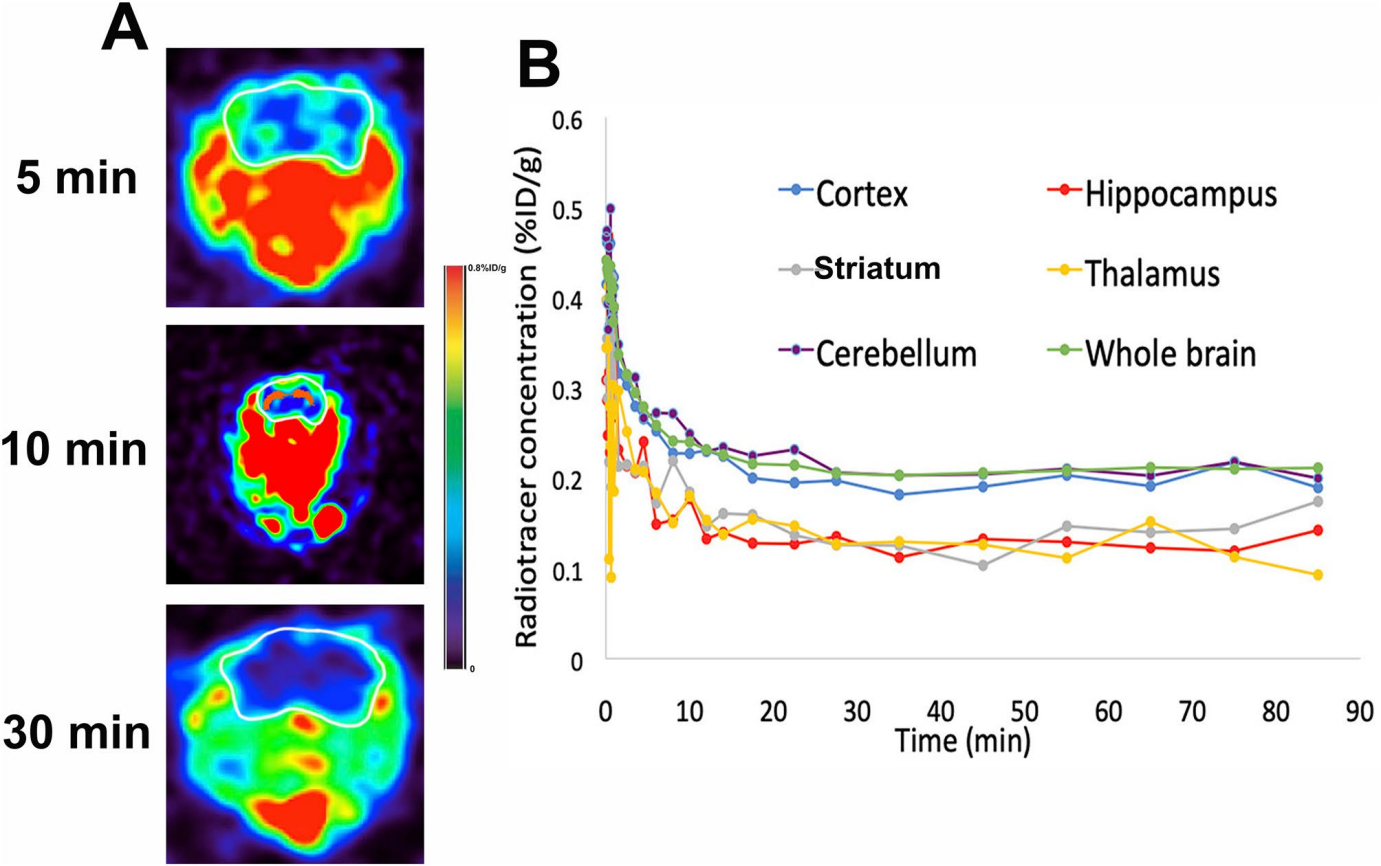
A representative axial view of a mouse (n = 4) injected with [^11^C]ERGO simultaneously with the start of 90 min dynamic acquisition in a microPET. (**A**) PET/CT image outlining the skull of the mouse at different timepoints; (**B**) the time-activity-curves (TAC) within different regions of the brain. *%ID/g *percentage of injected dose per animal weight.

To test the distribution of ERGO non-invasively in vivo, approximately 37 MBq/0.1 mL of [^11^C]ERGO probe was given to anesthetized 5XFAD mice (8-month-old, n = 4) in single bolus intravenous (I.V.) injection via the lateral tail vein. The administration of the probe was simultaneous with the start of a 90-min dynamic PET imaging, followed by subsequent CT scan. The PET images were normalized to the injected dose, and the time-activity-curves (TACs) of the mean activity within the ROIs were estimated for the entire duration of the scans. Within 5 min post I.V. injection, [^11^C]ERGO can be detected in the brain parenchyma (Fig. 4A, top panel). Approximately 10 min post I.V. injection, the radioligand was distributed in several areas of the brain (Fig. 4A, middle panel), and it appeared the probe still remained in the brain 30 min post radiotracer injection (Fig. 4A, bottom panel). Using an age-matched mouse brain template, the positron-emitted signal at different brain regions was quantified using AMIDE software. The data showed that the probe was distributed with a high concentration in the cerebellum and cortex (Fig. 4B). Nevertheless, the presence of the probe in other brain areas, such as the hippocampus, striatum, and thalamus, was also significant.

### Imaging LPS-induced neuroinflammation using [^11^C]ERGO radioligand

**Figure 5:**
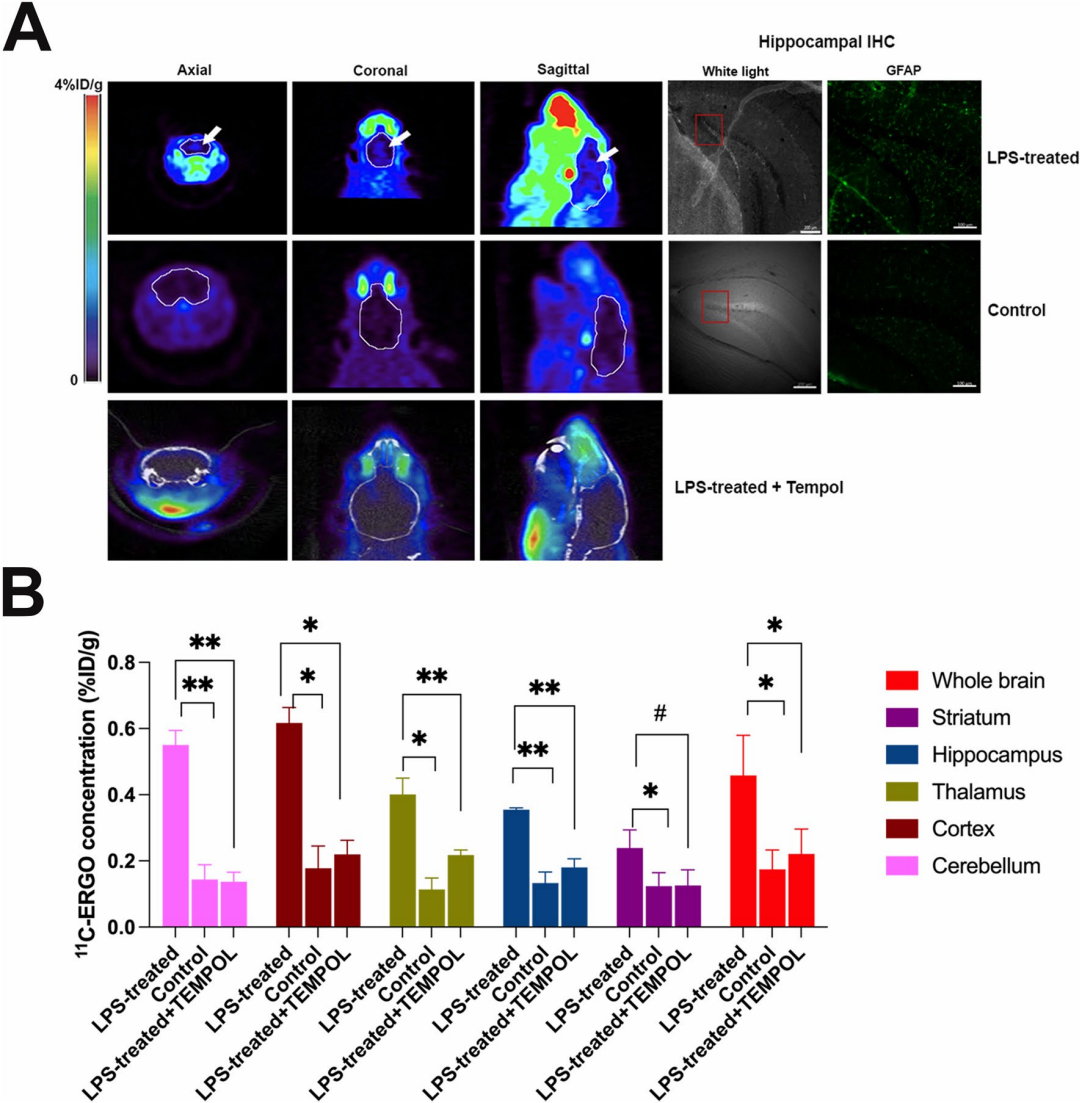
Imaging oxidative stress in the LPS-induced neuroinflammation mouse model using [^11^C]ERGO radioligand. (**A**) Representative view of the PET signal (white arrows) depicted from the brain of LPS-treated (n = 3) versus non-treated control mice (n = 3) and LPS-treated mice, co-injected with Tempol (n = 3). The immunohistochemical analysis of the hippocampal region using anti-GFAP antibodies. The observed fluorescent signal was indicated in red squares; (**B**) semi-quantitative analysis of the uptake in the subregions of the brains of LPS-treated versus control and LPS-treated + Tempol. *P < 0.05; **P < 0.005, ^#^P = 0.05.

The reported LPS mouse model of neuroinflammation in the past^40^ was reproduced for the validation of the specificity of the probe. Approximately 24 h after injection of WT mouse with LPS (5 mg/kg) via intraperitoneal injection, the animals showed signs of withdrawing and lacked grooming. The animals experienced noted body weight loss over 10% within a day after treatment. Further, histological analysis of the brain sections at the hippocampal region showed a significant upregulation of GFAP in the LPS-treated mice (Fig. 5A). The control nontreated WT (n = 3) and LPS-treated WT mice (n = 3) were injected with [^11^C]ERGO radioligand (20 MBq/0.1 mL) via I.V. injection. For the blocking study, another cohort of LPS-treated WT mice (n = 3) was injected with Tempol (4-hydroxy-2,2,6,6-tetramethyl piperidinoxyl) (0.2 mg/mL) prior to injection of the same dose of [^11^C]ERGO. Ten minutes after injection, each animal was imaged for a period of 30 min, followed by CT. Data shown in Fig. 5A indicated that ERGO was distributed and retained in the brain of LPS-treated mice resulting in higher signal intensity on the PET scans. The PET information was coregistered with CT and MRI using the MRI template of age-matched mice. When PET signal in each brain region was analyzed and compared (Supplementary data, Fig. S2), the data showed that the signal from LPS-treated mice was stronger than that from nontreated counterparts across every major region of the brain (Fig. 5B). The specificity of the probe was demonstrated in the blocking study; as co-injection of the [^11^C]ERGO probe with the excess amount of Tempol resulted in a significant reduction of the PET signal (Fig. 5).

### Whole-body biodistribution of [^11^C]ERGO radioligand

**Figure 6:**
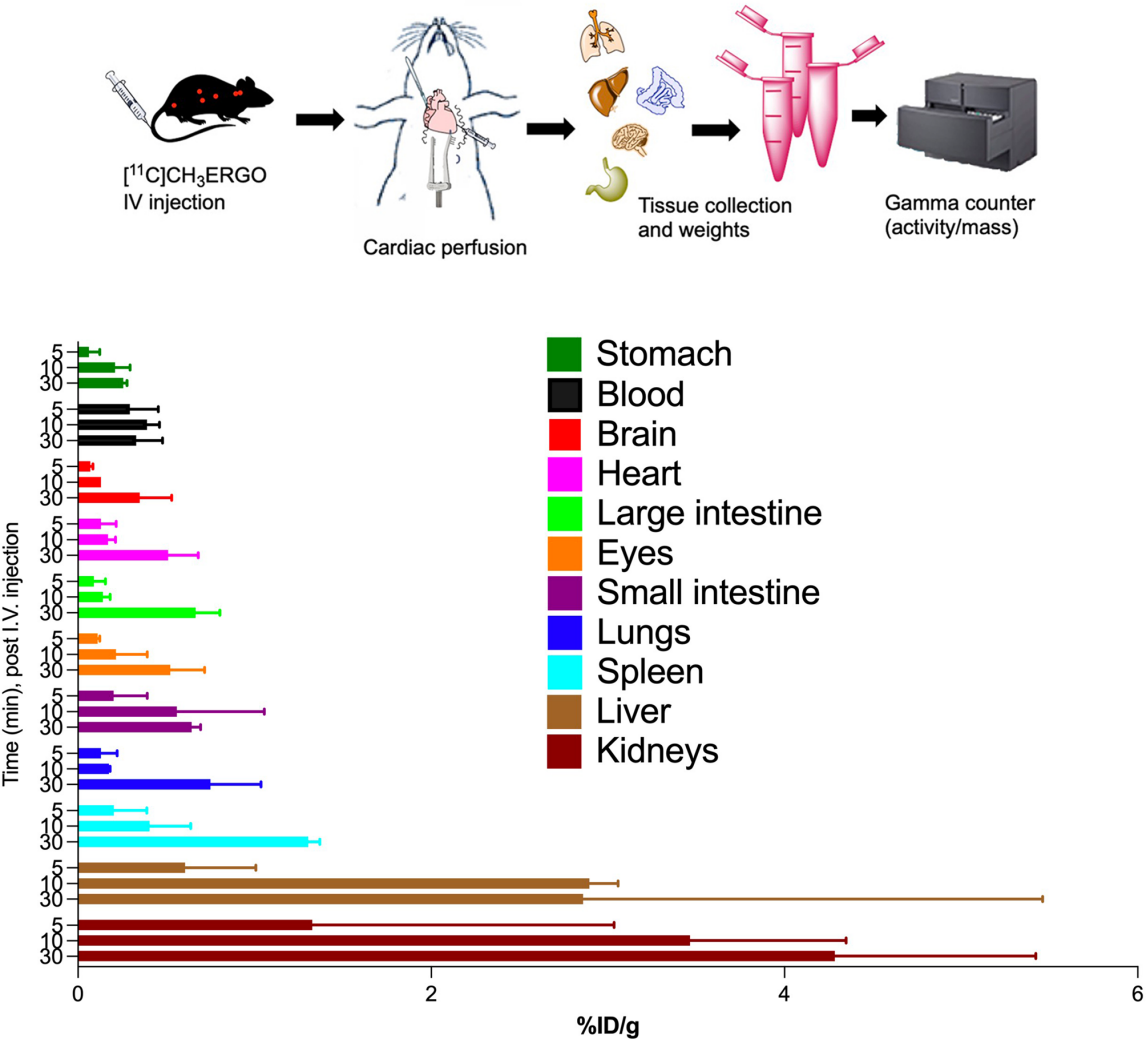
Ex-vivo cut-and-count biodistribution of I.V.-injected [^11^C]ERGO radioligand in WT mice. At 5 (n = 5), 10 (n = 5) or 30 (n = 6) min post I.V. injection, animals went through cardiac perfusion before tissues collections and counted. Tissue radioactivity was assessed and expressed as %ID/g.

Cohorts of WT mice were injected with [^11^C]ERGO probe, followed by cardiac perfusion 5 (n = 5), 10 (n = 5) and 30 (n = 6) minutes after injection before tissues were collected for activity counting. The data in Fig. 6 showed that ERGO was quickly distributed to every organs, including those have barriers such as brain (blood–brain barrier) and eyes (blood-retinal barrier) 5 min post I.V. injection. About 30 min after injection of [^11^C]ERGO, the concentration in the blood decreased (from 0.39 to 0.33%ID/g), at the same time compound continued to accumulate in the peripheral tissues, including the brain (from 0.13 to 0.35%ID/g), the eyes (from 0.11 to 0.52% ID/g), spleen (from 0.2 to 0.97% ID/g), small intestine (from 0.2 to 0.64%ID/g), heart (from 0.13 to 0.51%ID/g), kidneys (from 1.33 to 4.2%ID/g) and liver (0.61–2.86%ID/g).

## Discussion

One of the objectives of this probe development was to assess a number of synthetic pathways to ensure the probe would be designed in a way that minimized the potential alternation of the intrinsic chemical structure. Ideally, we sought to maintain an identical structure to ERGO. In this respect, the most logical approach focuses on the quaternary amine, which presents a convenient opportunity to insert the [^11^C]CH_3_ radioisotope through the use of an [^11^C]CH_3_ electrophile. Since ERGO comprises four distinct functional groups and each can be activated as a strong nucleophile at their respective pKa, so it is conceivable that each step of the chemistry may require prior protection and deprotection using orthogonal strategy. Further, incorporating these protecting groups is crucial to enhance the hydrophobicity of this water-soluble zwitterion molecule for organic solvents. Given that ERGO and histidine share an overall structural similarity; therefore, taking all of these observations into an account, the design suggests the adoption of either N- or C-terminal protected histidine as the starting material.

Multiple attempts were tried to synthesize the precursor **7**; in almost every attempt, two major issues were encountered. First, the product was so polarized that it proved to be challenging for purification using flash chromatography. Second, the protecting groups were sometimes so bulky that they obscured the next step in the process, or they were cleaved off. Therefore, the choice of a protecting group is crucial for the successful synthesis. As in this case, using an orthogonal strategy, the Fmoc group can be removed without affecting the Boc group in the following steps. Aside from protection, the Fmoc helps to enhance hydrophobicity and its UV active nature, enables reaction monitoring. After three functional groups were protected, the Boc from the N-terminal region was selectively removed using TFA in methylene chloride at − 78 °C to afford **4 (**Fig. 3). The reaction was carefully performed and monitored to ensure the *t*-Bu ester is tolerant to these reaction conditions. There was no sign that *t*-Bu ester was affected for an extension of up to 4 h. However, when the reaction was continued overnight, both the Boc and the *t*-Bu ester groups were removed completely. It is interesting to mention that when treating compound **4** with sodium cyanoborohydride during the reductive amination, the Fmoc group was also removed concomitantly. This was an unintentional but necessary process. Fabrication of an appropriate PET precursor requires that all protecting groups should have a Boc group, which can be removed simultaneously in mild acidic condition once after radioisotope labeling. This requires that the Fmoc group should be removed and replaced with a Boc group.

ERGO has multiple advantages when it comes to studying oxidative stress in the brain, given the molecule is very water soluble, ideal for the clinical formulation whether it is for I.V. injection or for oral usage. But the paradox is that hydrophilic molecules cannot cross the blood–brain barrier (BBB). A unique feature of ERGO compared to other antioxidants is that its distribution to the brain parenchyma is independent of the BBB, but rather mediated by OCTN1 receptors. Thus, it seems reasonably certain that there is a huge interest in assessing the protective role of ERGO in the brain^15,23,29^, particularly oxidative stress related to Alzheimer’s disease^40–42^. In clinical trials, either pure ERGO or mushrooms have been given to testing subjects for examining its effect on biomarkers of oxidative damage and inflammation^43–45^. So far, all ERGO biodistribution and pharmacokinetics analysis rely on the indirect analysis of blood and urine samples^43^.

The availability of the probe enables dynamic imaging to assess the biodistribution and kinetics of ERGO at any target of interest. Further, it also provides information for mapping OCTN1 receptors. For instance, the data in Fig. 4 showed that within 5 min post I.V. injection, the compound started to present in the brain. At 10 min, ERGO is available almost in every region of the brain. The cortex and cerebellum had the highest regional uptake of the radiotracer compared to the hippocampus, striatum, and thalamus at every timepoint along with the TAC (Fig. 4B). This observation is consistent with a past study that showed ERGO was found in a large quantity in the cerebellum than in the rest of the brain across different species, including mice, rats, guinea-pigs, cats, and sheep^46^. The presence and variable distribution of ERGO in the brain, with high concentrations in the central nervous system suggesting the possibility of its role in the central regulatory function. In addition, the observation of ERGO presence in the hippocampus of 5XFAD mouse supports a focus of future studies to assess the implications of ERGO in Alzheimer’s disease. In the recent past, it has been reported the inhibition of Abeta-induced toxicity by ERGO in the transgenic Caenorhabditis elegans model^47^. With the availability of this probe, similar studies in higher organisms are on the horizon.

The retention of the [^11^C]ERGO probe depicted by strong signal in the brains of 5XFAD mice during PET scans (Fig. 4) was presumed due to chronic neuroinflammation occurs in Alzheimer’s disease^48^. As a stronger demonstration of the specificity of the probe, we used LPS-induced mice as a model of acute neuroinflammation. LPS is a molecular motif structurally similar amongst gram-negative bacteria that is recognized by the innate immune system and results in pro-inflammatory cytokine release mediated by toll-like receptor 4 (TLR-4)^49^. We have previously shown that injection of LPS (I.V.) resulted in severed neuroinflammation that breached the blood–brain barrier even at a nonlethal, low dose (< 3 mg/kg)^50^. Following observations of lethality at a dose greater than 3 mg/kg combined with recent literature^51^, we decided to inject LPS into the peritoneum to prevent a robust inflammatory response. The 30-min PET scans (Fig. 5) suggested that ERGO was retained in the brain, likely acting as an adaptive antioxidant. The signal intensity of the LPS-treated mice was significantly higher than that found in control mice. And it is apparent that the specific distribution and retention of the probe is ROS-dependent, not because of the LPS-induced BBB opening. Since pretreatment of LPS mice with Tempol, a general-purpose antioxidant known for scavenging a wide range of ROS and reactive nitrogen species^52^, resulting in a remarkable reduction in the [^11^C]ERGO signal across all the brain regions (Fig. 5).

Similar to the brain study, data from the whole-body biodistribution showed that 5 min post-injection, ERGO started to present in the peripheral tissues, as the levels in the blood reduced significantly (Fig. 6). Five minutes later, the compound is distributed mostly in every tissue, as a note, the level of ERGO is very high in the eyes and the small intestines. This in vivo observation is consistent with the past data, which also reported ERGO primarily concentrates in those tissues^1^. Given the photo-oxidative process, the eyes are also susceptible to ROS and oxidative stress, and subsequent inflammatory conditions^53^. Based on the data shown in Fig. 5 (coronal and sagittal information), it seems likely this [^11^C]ERGO probe could detect the inflammation (LPS-treated) and response (after treating with Tempol) to the treatment in the eyes. More investigation is on the way in our laboratory since we think the molecular mechanisms underlying these differences could be of considerable biomedical importance.

Different from the in vivo PET imaging information, the biodistribution data showed that over the thirty minutes after administration, the ERGO continues to accumulate in most of the tissues, including the brain (Fig. 6). This discrepancy may be due to blood flow effects that are present only in the microPET data. Any signal due to ERGO presence in the blood is absent in the biodistribution data since perfusion of the vasculature was performed prior to measurement of the radioactivity in excised tissues. This observation could be explored in future studies through kinetic modeling of the dynamic PET data to better separate tissue uptake from blood flow effects.

## Conclusion

In summary, a new [^11^C]ERGO PET radioligand has been synthesized to facilitate in vivo, non-invasive and real-time imaging of the biodistribution of ERGO. The development of a probe with an identical structure of ERGO is a significant advantage to our design. As such, all pharmacokinetics and biodistribution shown in this work are expected to reflect those of native compounds. Further, the availability of the PET radioligand enables the performance of longitudinal investigations in the same animals. Thus, differences between animals due to inter-individual variations can be controlled for^54^. Overall, we anticipate that this probe will pave the way for the integration of molecular imaging with food biomarkers and biomedical research. The probe provides an emerging capability that will benefit the ERGO research community.

## Materials and methods

TEMPOL was acquired from Tocris Bioscience (Fisher Scientific). Animal experiments were conducted per the guidelines established by the Vanderbilt University’s Institutional Animal Care and Use Committee (IACUC) and the Division of Animal Care. The performed work was approved by Vanderbilt IACUC with an extended protocol, M1700044-01. In addition, all of the works involving live animals were compliant with the ARRIVE guidelines. In a typical imaging procedure, anesthetized mice received 2% of isoflurane via inhalation, supplied with oxygen using a precision vaporizer.

### General synthesis

All commercially available reagents and solvents were used as received. L-(+)-Ergothionene was purchased from Cayman Chemicals. Reactions were montitored by a Agilent LC/MS 1260 Infinity II. Products were purified using a Telodyne Combiflash Rf automated purification instrument using normal phase or reverse phase. NMR spectra were recorded on a 400 MHz Bruker AVANCE 400 equipped with a 9.3 T Oxford Magnet or 600 MHz Bruker AVIII console equipped with a 14.1 T Bruker Magnet. 1H NMR chemical shifts were referenced to the residual solvent signal; 13C NMR chemical shifts were referenced to the deuterated solvent signal. Data are presented as follows: chemical shift d (ppm), multiplicity (s = singlet, d = doublet, dd = doublet of doublet, t = triplet, m = multiplet).

### Synthesis of the PET precursor (Fig. 3)

#### *N*^τ^-(((9*H*-Fluoren-9-yl)methoxy)carbonyl)-*N*^α^-(*tert*-butoxycarbonyl)-*L*-histidine 2

To a stirring solution of histidine amino acid **1** (1.0 g, 3.9 mmol), FMOC-succinimide (1.45 g, 4.3 mmol), and DIPEA (0.605 g, 4.7 mmol) in methylene chloride (50 mL) and stirred overnight. Reaction was diluted with water and methylene chloride. The product was extracted 3 × with methylene chloride. The organic layers were combined, washed with 1 M HCl, saturated sodium bicarbonate, brine, dried over sodium sulfate, filtered and concentrated under reduced pressure to provide a desired product with 60% yield.

^1^H CDCl_3_ (400.13 mHz): 8.26 (d, J = 1.2 Hz, 1H); 7.79 (d, J = 7.6 Hz, 2H); 7.56 (d, J = 7.6 Hz, 2H); 7.43, (t, J = 7.2 Hz, 2H); 7.34 (t, J = 7.2 Hz, 2H); 7.24 (s, 1H); 5.51 (d, J = 6.0 Hz, 1H); 4.70 (dd, J_1_ = 7.2 Hz, J_2_ = 1.6 Hz, 2H); 4.56 (t, J = 7.6 Hz, 1H); 4.35 (t, J = 6.8 Hz, 1H); 3.32 (dd, J_1_ = 14.8 Hz, J_2_ = 2.8 Hz, 1H); 3.20 (dd, J_1_ = 14.4 Hz, J_2_ = 5.2 Hz, 1H); 1.47 (s, 9H). ^13^C CDCl_3_ (100.6 mHz): 172.9, 168.6, 155.4, 148.1, 142.7, 141.5, 137.5, 128.4, 127.6, 124.9, 120.5, 115.6, 80.0, 70.6, 53.6, 52.8, 46.6, 29.9, 28.5.

#### (9*H*-Fluoren-9-yl)methyl(*S*)-4-(3-(*tert*-butoxy)-2-((*tert*-butoxycarbonyl)amino)-3-oxopropyl)-1*H*-imidazole-1-carboxylate 3

To a stirring solution of **2** (3.90 mmol) in methylene chloride (50 mL) and tert-butanol (10 mL)at 0 C was added: EDC-HCl (744 mg, 3.9 mmol), and DMAP (95 mg, 0.78 mmol. This solution was allowed to warm up to r.t. and stirred overnight. Reaction was diluted with water and methylene chloride. The product was extracted 3 × with methylene chloride. The organic layers were combined, washed with 1 M HCl, saturated sodium bicarbonate, brine, dried over sodium sulfate, filtered and concentrated under reduced pressure to provide the desired product (12% yield).

^1^H CDCl_3_ (400.13 mHz): 7.99 (s, 1H); 7.81 (d, J = 7.6 Hz, 2H); 7.58 (d, J = 7.6 Hz, 2H); 7.48, (t, J = 7.2 Hz, 2H); 7.35 (t, J = 7.2 Hz, 2H); 7.17 (s, 1H); 5.03 (m, 1H); 4.72 (dd, J_1_ = 6.4 Hz, J_2_ = 2.0 Hz, 2H); 4.20 (m, 1H); 3.03 (m, 2H); 1.44 (s, 9H).

#### (9*H*-Fluoren-9-yl)methyl (*S*)-4-(2-amino-3-(*tert*-butoxy)-3-oxopropyl)-1*H*-imidazole-1-carboxylate 4

To a stirring solution of **3** (3.90 mmol) in methylene chloride at − 78 °C was added TFA (5 mL) dropwise. This solution was allowed to warm up to 0 °C and stirred until completion. The solution was then neutralized with sodium bicarbonate and diluted with water and methylene chloride. The product was extracted 3 × with methylene chloride. The organic layers were combined, washed with brine, dried over sodium sulfate, filtered and concentrated under reduced pressure to provide the desired product with 63% yield.

^1^H CDCl_3_ (400.13 mHz): 7.99 (s, 1H); 7.77 (d, J = 7.6 Hz, 2H); 7.57 (d, J = 6.8 Hz, 2H); 7.41, (t, J = 6.0 Hz, 2H); 7.33 (t, J = 7.6 Hz, 2H); 7.14 (s, 1H); 5.03 (m, 1H); 4.59 (m, 2H); 4.18 (m, 1H); 3.05 (m, 2H); 1.38 (s, 9H).

#### *Tert*-Butyl *N*^α^,*N*^α^-dimethyl-*L*-histidinate 5

To a stirring solution of **4** in MeOH (20 mL) was added NaBH_3_CN (362 mg, 5.8 mmol) and CH_2_O (37% in H20, 702 mg, 23.4 mmol). The reaction was capped and stirred for 2 h. The resulting solution was then concentrated via rotovap. The crude oil was purified by reverse phase chromatography. The product was verified by LC/MS and used as is in the next step with a 82% yield. The Fmoc group was removed in this step through interaction with the NaBH_3_CN to provide the desired product.

^1^H D_2_O (400.13 mHz): 7.47 (s, 1H); 6.83 (s, 1H); 4.79 (m, 1H); 4.29 (m, 2H); 2.58 (s, 6H); 1.24 (s, 9H).

#### *Tert*-Butyl (*S*)-2-(dimethylamino)-3-(2-thioxo-2,3-dihydro-1*H*-imidazol-4-yl)propanoate 6

To a stirring solution of **5** (31.5 mg, 0.13 mmol) in water (2 mL) and diethyl ether (2 mL) was added sodium bicarbonate (65 mg, 0.78 mmol), and phenylchloro thionoformate (24.7 mg, 0.14 mmol) and stirred overnight. Reaction was diluted with water and diethyl ether. The product was extracted 3 × with diethyl ether. The organic layers were combined, washed with brine, dried over sodium sulfate, filtered and concentrated under reduced pressure. The resulting oil was then redissolved in MeOH (5 mL) and triethylamine (55 μL) was added. This solution was stirred overnight. The resulting solution was then concentrated under reduced pressure. Product was purified by reverse phase chromatography to provide a final product (16.0 mg) 45% yield. And the product was immediately used for the next step.

#### *Tert*-Butyl (*S*)-4-(3-(*tert*-butoxy)-2-(dimethylamino)-3-oxopropyl)-2-((*tert*-butoxycarbonyl)thio)-1*H*-imidazole-1-carboxylate 7

To a stirring solution of **6** (16.0 mg, 0.066 mmol) in methylene chloride (10 mL) was added: Boc anhydride (32 mg, 0.145 mmol), and DIPEA (19 mg, 0.145 mmol) and stirred over the course of 48 h. The resulting solution was concentrated and purified by flash chromatography (0–50% CH2Cl2/(20%MeOH/CH2Cl2)) to afford the precursor **7** (15.0 mg) with 48% yield.

^1^H CDCl_3_ (600.13 mHz): 7.16 (s, 1H); 3.40 (m, 1H); 2.86 (dd, J_1_ = 14.4 Hz, J_2_ = 8.4 Hz, 1H); 2.72 (dd, J_1_ = 14.4 Hz, J_2_ = 6.6 Hz, 1H); 2.28 (s, 6H); 1.48 (s, 9H); 1.37 (s, 9H); 1.32 (s, 9H). ^13^C CDCl_3_ (150.9 mHz): 170.9, 165.3, 146.7, 139.9, 135.1, 119.8, 86.8, 85.8, 81.2, 67.3, 41.8, 29.8, 28.6, 28.3, 28.2, 27.9, 22.3, 22.0.

MS: calculated: 471.2403, detected: 471.1845.

### [^11^C]ERGO radiotracer synthesis

The [^11^C]CO_2_ was made by irradiating a target filled with nitrogen and 1% oxygen gas with protons. The [^11^C]CO_2_ was then trapped on nickel Shimalite with molecular sieves at room temperature. The [^11^C]CO_2_ was then converted to [^11^C]CH_4_ by heating the trapped [^11^C]CO_2_ to 400 °C in the presence of hydrogen gas. The [^11^C]CH_4_ was then released from the nickel Shimalite at 400 °C and isolated on molecular sieves at -75 °C. The [^11^C]CH4 was then converted to [^11^C]MeI via a recirculation through gaseous iodine at ~ 720 °C, with the [^11^C]MeI being trapped on Porapak N with each cycle. The [^11^C]MeI was then released from the Porapak N by heating with a gentle flow of helium that is passed through an AgOTf impregnated column at ~ 200 °C to convert the [^11^C]MeI to [^11^C]MeOTf. This [^11^C]MeOTf was bubbled into a solution of precursor in 250 μL acetonitrile at − 10 °C. After the activity transfer was complete, the reaction mixture was heated to 80 °C for 2 min. At this time, hydrochloric acid (6 M, 250 μL) was added, and the reaction mixture was heated at 70 °C for 5 min, cooled to room temperature, and diluted with water (1 mL). The reaction mixture was passed through an ion-retardation resin (Ag11–A8, 3 g) into the product vial, and the resin was rinsed with water (5 mL) into the product vial. The product was then transferred to the final vial, and an aliquot was removed for quality control analysis.

The radiochemical purity and the identity of the [^11^C]ERGO were characterized using an analytical HPLC system, equipped with a UV absorption detector (λ = 254 nm) and a radio-detector (Bioscan Flow-Count). The chromatography setup included a SeQuant ZIC-HILIC 150 × 4.6 mm with a typical mobile phase of acetonitrile (75%) in water at a flow rate of 1 mL/min. The identity of the [^11^C]ERGO was confirmed by comparing the retention time with co-injected and unlabeled ERGO along with the gamma peak. The molar activity of the radioligand is 690 TBq/mmol.

### PET/CT data processing and analysis

The dynamic acquisition was divided into twelve 5 s frames, four 60 s frames, five 120 s frames, three 5 min frames, and six 10 min scans. The data from all possible lines of response (LOR) were saved in the list mode raw data format. The raw data were then binned into 3D sinograms with a span of 3 and ring difference of 47. The images were reconstructed into transaxial slices (128 × 128 × 159) with voxel sizes of 0.0815 × 0.0815 × 0.0796 cm^3^, using the MAP algorithm with 16 subsets, 4 iterations, and a beta of 0.0468. For anatomical co-registration, immediately following the PET scans, the mice received a CT scan in a NanoSPECT/CT (Mediso, Washington DC) at an X-ray beam intensity of 90 mAs and x-ray peak voltage of 45 kVp. The CT images were reconstructed into 170 × 170 × 186 voxels at a voxel size of 0.4 × 0.4 × 0.4 mm^3^. The PET/CT images were uploaded into Amide software (www.sourceforge.com), co-registered to an MRI template made in-house, and volumetric regions-of-interest were drawn around the cortex, hippocampus, striatum, thalamus, and cerebellum in addition to the whole brain. The PET images were normalized to the injected dose, and the time-activity-curves (TACs) of the mean activity within the ROIs were estimated for the entire duration of the scans.

### Static PET scan

The mice were injected with ~ 20 MBq/0.1 mL 11C-ERGO via the tail vein and returned to their cages for 10 min. Then they were anesthetized with 2% isofluorane and imaged in an Inveon microPET (Siemens Pre-clinical, Knoxville, TN, USA) for 30 min in static mode followed by a CT scan using a NanoSPECT/CT (Mediso, Washington DC) at an X-ray beam intensity of 90 ms and X-ray voltage of 45 kVp. PET images were reconstructed using the iterative MAP reconstruction algorithm with 18 iterations and a beta smoothing value of 0.001 into 128 × 128 × 95 slices with a voxel size of 0.388 mm × 0.388 mm × 0.796 mm. The CT images were reconstructed into 170 × 170 × 126 slices at a voxel size of 0.4 × 0.4 × 0.4 mm^3^. The PET and CT images were co-registered using the imaging tool AMIDE.

### Cardiac perfusion procedure and tissue collection

At 5 or 10 min post injection, anesthetized mice were laid on the shallow tray filled with crushed ice and the thoracic cavity was accessed using a scalpel after making 5–6 cm mid-line incision from the abdominal area. After careful separation of the liver from the diaphragm, the thoracic opening was stabilized with a retractor. Perfusion was performed as we described in the past^55–57^. Following removal of air bubbles, approximately 30 mL (pH7.4) of ice-cold PBS buffer is slowly injected in the left ventricle toward the ascending aorta using a 25-G syringe while the right atrium was snipped off using a curved point squeeze snip scissors to facilitate drainage of the systemic venous return. Then, 30 mL of 4% paraformaldehyde (PFA, pH 7.4) was perfused. When completed, tissues were harvested and weighed before counting using the automatic gamma counter (Hidex).

### Animal models

5XFAD and control C57BL/6J mice were maintained at Vanderbilt University under standard conditions, in a 12-h light/dark cycle, and with free access to food and water. The 5XFAD mice over express both mutant human amyloid precursor protein (APP) and presenilin 1 (PS1), correlating with high burden and accelerated accumulation of the Aβ. A colony of 5XFAD transgenic mice obtained from Jackson Laboratories was maintained by crossing 5XFAD mice with a WT C57BL/6J strain. The 5XFAD mice were maintained as heterozygous.

The mouse model of LPS-induced neuroinflammation was developed based on past reports^50,51^. The LPS derived from *Escherichia* coli O111:B4 (Sigma Aldrich, St Louis, MO) was formulated in sterilized dd. water and given a high dose of LPS formulation (5 mg/kg) through intraperitoneal injection 24 h before PET imaging. This high dose would result in approximately 12–13% body weight loss over the course of 24 h. After PET imaging, animals were sacrificed, and the brains were collected for histology analysis.

### Immunohistochemistry

Brains embedded in OCT were cut into sagittal sections (10 µm) using a Tissue-Tek cryostat and mounted onto charged glass slides. Prior to staining, slides were washed with PBS (10 min); then, they were treated with blocking buffer (5% normal goat serum, 0.2% Triton X-100, 0.5% bovine albumin in PBS) for 1 h at room temperature. The treated sections were then incubated overnight at 4 °C with primary anti-GFAP antibody (1:100 dilution, Biolegend San Diego, CA, USA). Slides were washed with PBS (3×) for 10 min each, the sections were subsequently incubated with secondary antibody goat anti-mouse Alexa Fluor 488 (1:200 dilution, Thermo Fisher Scientific, Carlsbad, CA, USA) for 30 min at room temperature. The sections were then washed with PBS twice for 10 min and once for 30 min, and cover slipped with an antifade mounting medium (Vector Laboratories, Burlingame, CA, USA) before observation under a fluorescence microscope.

### Statistical analysis

Unpaired t test was used to compare the mean signal (%ID/g) difference between two independent subjects. A p value of ≤ 0.05 was considered as a statistically significant difference.

## Supplementary Information

Supplementary Information 1.
