# Supplementary material for: A novel antioxidant ergothioneine PET radioligand for in vivo imaging applications
Source: Sci Rep. 2021 Sep 16;11:18450. doi: 10.1038/s41598-021-97925-w (PMC8446031; doi:10.1038/s41598-021-97925-w)
Supplement: Supplementary file 1 — Supplementary Information 1. [file 41598_2021_97925_MOESM1_ESM.docx]

**Supplementary data**

**A novel antioxidant Ergothioneine PET radioligand for *in vivo* imaging applications**

William J. Behof ^1,2^, Clayton A. Whitmore ^1,2^, Justin R. Haynes ^1,2^, Adam J. Rosenberg ^1,2^, Mohammed N. Tantawy ^1,2^, Todd Peterson^1,2^, Fiona E. Harrison ^3^, Robert B. Beelman ^4^ and Wellington Pham* ^1,2,5,6,7,8,9,10^

1 Vanderbilt University Institute of Imaging Science, Vanderbilt University Medical Center, Nashville, TN 37232, USA

2 Department of Radiology and Radiological Sciences, Vanderbilt University Medical Center, Nashville, TN 37232, USA

3 Department of Medicine, Diabetes, Endocrinology & Metabolism, Vanderbilt University Medical Center, Nashville, TN 37232, USA

4 Department of Food Science, Center for Plant and Mushroom Foods for Health, Penn

State University, University Park, PA 16802, USA

5 Department of Biomedical Engineering, Vanderbilt University, Nashville, TN 37235,

USA

6 Vanderbilt Brain Institute, Vanderbilt University, Nashville, TN 37232, USA

7 Vanderbilt Ingram Cancer Center, Nashville, TN 37232, USA

8 Vanderbilt Institute of Chemical Biology, Vanderbilt University, Nashville, TN 37232,

USA

9 Vanderbilt Institute of Nanoscale Science and Engineering, Vanderbilt University, Nashville, TN 37235, USA

10 Vanderbilt Memory and Alzheimer’s Center, Vanderbilt University Medical Center, Nashville, TN 37212, USA

*Correspondence: [wellington.pham@vumc.org](mailto:wellington.pham@vumc.org)


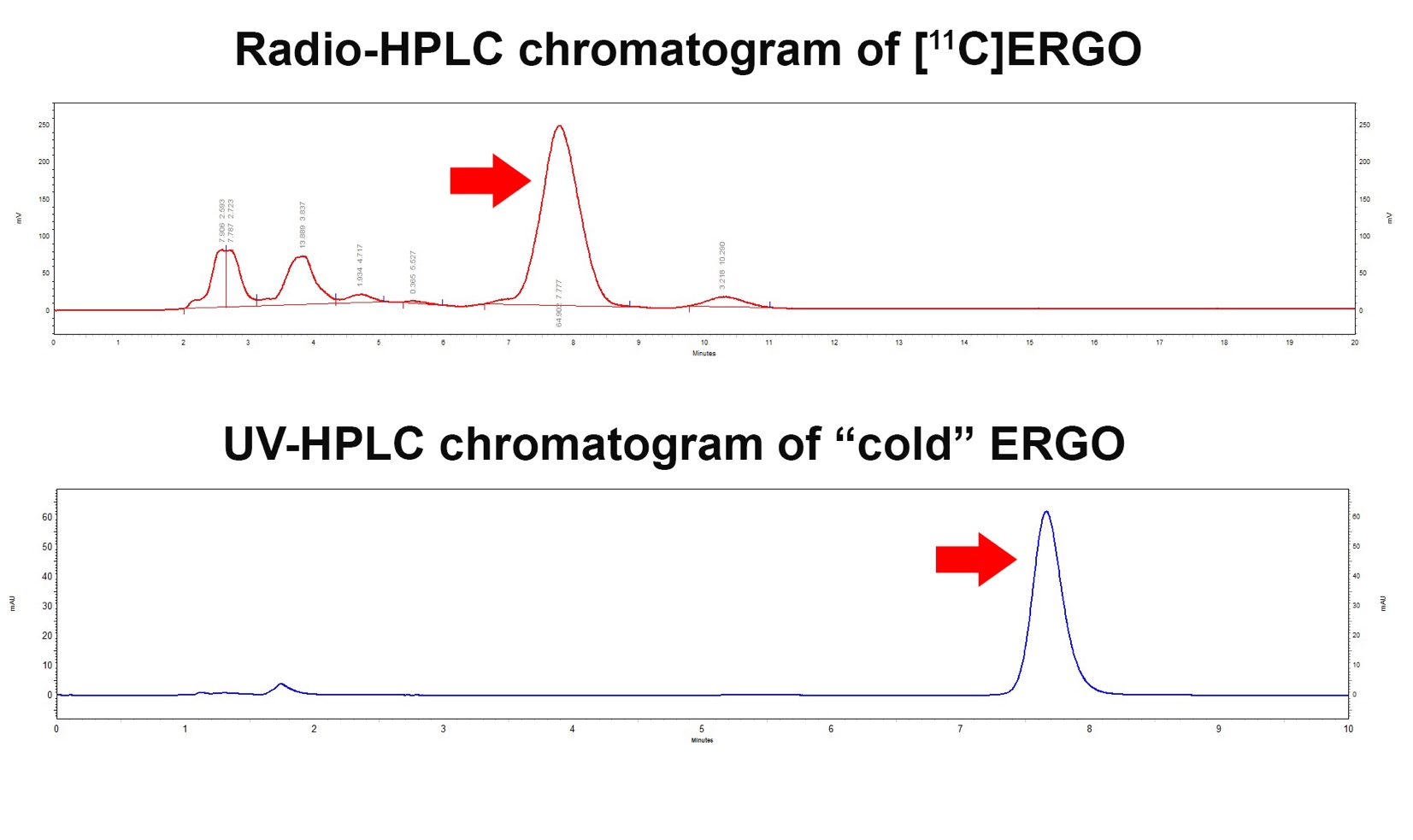


**Figure S1.** Confirmation of the product of [^11^C]labeling by comparing the HPLC elution time of [^11^C]ERGO with that from the “cold” compound via the UV-HPLC chromatogram.


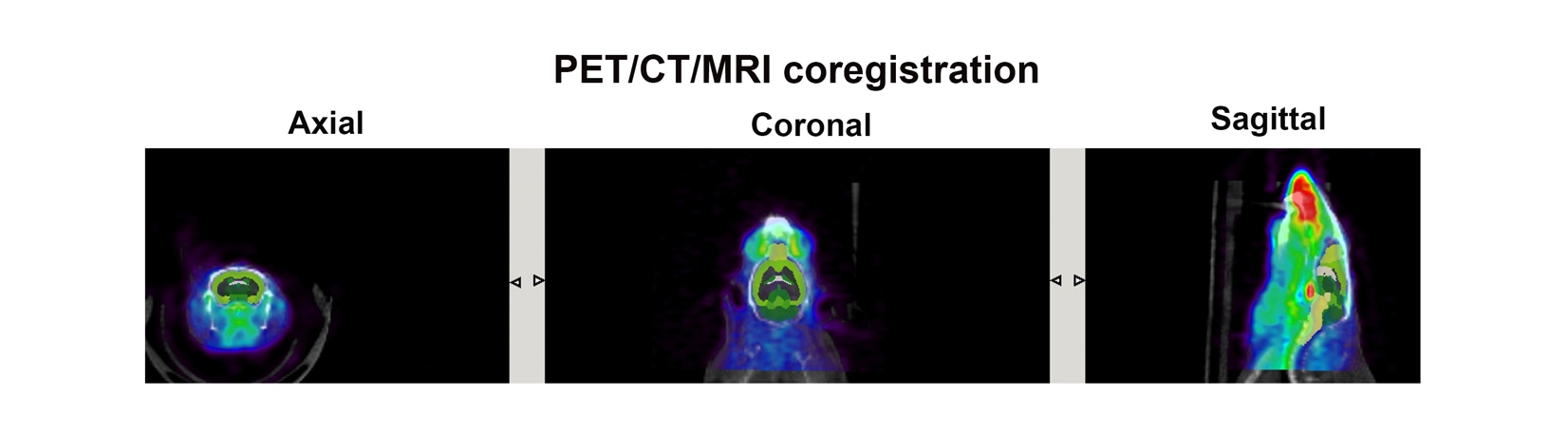


**Figure S2.** Representative data to demonstrate the coregistration of PET, CT, and MRI for the analysis of PET signals in subregions of the brain.
